# Aging microenvironment in osteoarthritis focusing on early-stage alterations and targeted therapies

**DOI:** 10.1038/s41413-025-00465-6

**Published:** 2025-10-10

**Authors:** Yifan Dang, Yuhang Liu, Bingjun Zhang, Xiaoling Zhang

**Affiliations:** 1 Department of Orthopedic Surgery, Xinhua Hospital Affiliated to Shanghai Jiao Tong University School of Medicine (SJTUSM), Shanghai, PR China; 2 Collaborative Innovation Centre of Regenerative Medicine and Medical BioResource Development and Application Co-constructed by the Province and Ministry, Guangxi Medical University, Nanning, Guangxi PR China; 3 National Facility for Translational Medicine, Shanghai, PR China; 4 Department of Orthopaedics, Shanghai Sixth People’s Hospital Affiliated to Shanghai Jiao Tong University School of Medicine, Shanghai, PR China

**Keywords:** Bone, Pathogenesis

## Abstract

Osteoarthritis (OA) is one of the most common degenerative and age-related diseases in joints, which affects 654 million people worldwide. Current therapies could not fundamentally reverse the pathologic process of OA due to the complex pathogenesis. Although OA mechanisms have been investigated on a large scale over the past decade, the OA pathology correlated with aging-associated changes is still largely unrevealed. Therefore, in-depth analysis of the aging microenvironment and aging-related molecular mechanisms in OA may offer additional strategies for clinical prevention and treatment. In this review, we discuss the potential pathogenesis of OA in light of aging-associated changes and summarize three main components of the aging microenvironment of the OA joint: immune homeostatic imbalance, cellular senescence, and stem cell exhaustion, which could be induced by aging and further exacerbate OA progression. Additionally, it is emphasized that immune homeostatic imbalance appears before established OA, which occurs in the early stage and is the therapeutic window of opportunity for better clinical outcomes. Importantly, we evaluate recent therapeutic targets and promising interventions against these components, as well as the challenges and prospects for precise and individualized therapies of OA patients, which we believe would guide the construction of novel combined strategies targeting aging-related factors against OA for better treatments in the future.

## Introduction

Aging is a serious global social issue and the predominant cause of many aging-related diseases. According to the WHO, 22% of the world’s population (roughly 2 billion people) will be over the age of 60 by 2050. Osteoarthritis (OA) is the most common joint disease related to the elderly, characterized by the irreversible loss of cartilage, formation of osteophytes, infiltration of the synovial membrane, and subchondral bone remodeling, involving the entire joint structure.^1^ However, the current therapies are unable to efficiently cure the disease or reverse cartilage degeneration, leading to a huge economic and social burden worldwide.

Previous studies have identified low-grade and chronic inflammation-primarily triggered by cartilage matrix degradation and mechanical stress as a key driver of OA.^2^ The inflammation is primarily influenced by the innate immune system, including Damage-associated molecular pattern (DAMP)-Toll-like receptors (TLR) signaling, complement activation, and immune cells like macrophages and mast cells, leading to elevated pro-inflammatory mediators in the joint microenvironment, such as cytokines, chemokines, growth factors, adipokines, prostaglandins, leukotrienes, nitric oxide, and neuropeptides, which further trigger synovitis and exacerbate OA progression.^3,4^ Synovitis, present throughout OA, is triggered by immune cells such as macrophages, T lymphocytes, and B lymphocytes, which can amplify the inflammatory response and cartilage damage.^4^ Besides, metabolic dysregulation and cytokine-mediated damage to chondrocytes also leads to OA, represented by changes in mitochondrial dynamics, cellular senescence, etc.^5^ Stem cells, the source of chondrocytes, are also implicated in OA pathology, and recent advances highlight their therapeutic potential.^6^ However, aging-related changes in synovium, chondrocytes, and stem cells, particularly in early OA, remain underexplored.

OA arises from diverse origins, including aging and injury. In age-related OA, mitochondrial dysfunction and oxidative stress induce cellular senescence in joint tissues.^7^ Tao et al. identified senescent cells in both bulk and single-cell transcriptome of 602 samples from 52 senescence transcriptome datasets via machine learning program senescent cell identification. The data showed that mitochondria and redox reactions in mesenchymal stem cell or stromal cell clusters are significantly different between senescent and non-senescent samples, and ribosomal function decline, a central pathway for senescence, showed difference in senescent and non-senescent CD4^+^ T cell clusters. What’s more, they distinguished aged or damaged tissues from their controls with high significance including OA chondrocytes. These data strongly supported that aging could lead to alterations of stem cells, immune cells and chondrocytes via a large-scale analysis of single cell sequencing data.^8^ Key biomarkers such as Fibroblast activation protein (FAP), Zinc Finger E-box Binding Homeobox 1 (ZEB1), and Sirtuin 6 (Sirt6) influence chondrocyte aging and OA progression.^9^ Specific ablation of Sirt6 in chondrocytes could exacerbate osteoarthritis via exaggerating chondrocyte senescence.^10^ Notably, senescence can spread locally via extracellular vesicles, as seen in aged macrophages promoting tissue-wide aging.^11,12^ Thus, senescence likely affects various cell types in the OA microenvironment beyond chondrocytes. Understanding these aging-related cellular changes is essential for advancing OA research, yet they remain insufficiently explored.

**Fig. 1:**
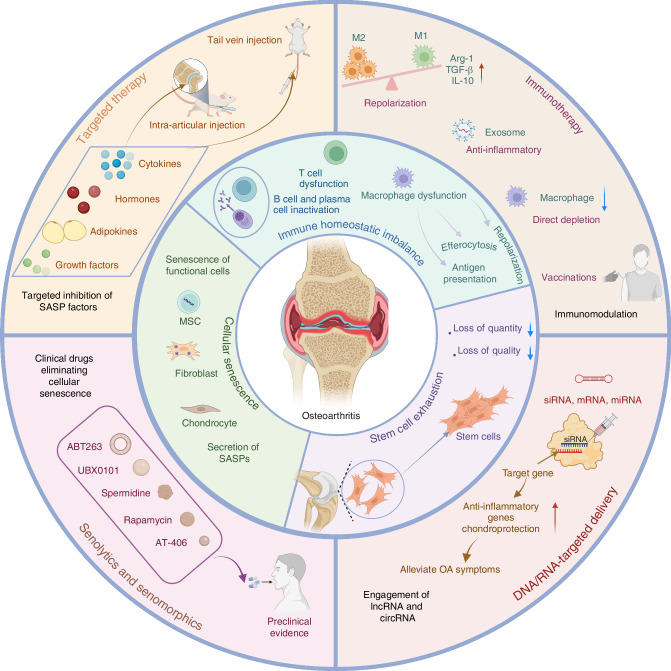
Aging microenvironment in OA joint. The alteration of aging microenvironment contributes to the onset and progression of OA, which consists of immune homeostatic imbalance, cellular senescence and senescence-associated secretory phenotype (SASP), and stem cell exhaustion, leading to various downstream changes of molecules and signaling pathways. Meanwhile, current treatments and promising strategies for prevention of OA are summarized

With the aging population increasing gradually, the elimination of senescent cells has emerged as an active direction in the research field of aging-related diseases. In the past decade, researchers have devoted significant efforts to this field, and an increasing number of senolytics and senomorphics have been designed and produced based on profound investigations of mechanisms of cellular senescence. Among the very few reviews on the topic of OA aging microenvironments, we noticed that a comprehensive summarization is still absent in this field, especially from the perspective of the relevant cells influenced by aging in OA. In this review, we will first introduce the major components affected by aging in OA, including immune homeostatic imbalance, senescent cells, and stem cell exhaustion, which we termed as the aging microenvironment of OA. Following that, we will highlight the functional alterations triggered by aging and related potential mechanisms leading to OA. Afterwards, we will summarize state-of-the-art design strategies for the development of application-oriented targeted therapies (such as immunotargeted therapies, senolytics, and stem cell-based treatments), demonstrating how modulations of aging microenvironment can be designed and exploited as an efficient strategy to treat OA. Finally, we will provide an outlook on future directions and challenges in the emerging research field of intercellular crosstalk in the aging microenvironment of OA. The review herein will provide researchers with useful information on the acknowledgment of aging-related alterations in OA pathology, as well as clues in selecting appropriate targets for novel precise therapy for the early stage of OA (Fig. 1).

### Immune homeostatic imbalance in aging microenvironment of OA

**Fig. 2:**
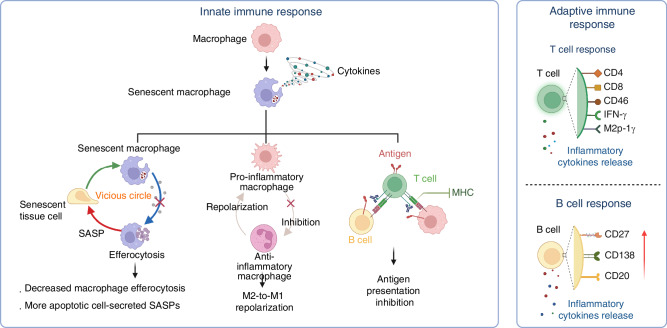
Immune homeostasis imbalance in OA joints. Immune homeostasis is composed of immune cells and related cytokines, which are mainly manifested by innate immune response and adaptive immune response. In the early stage of OA, immune cells differentiate into various phenotypes and exert functions via secretion of cytokines and chemokines, resulting in complex cascades and disruption of immune homeostatic balance

According to long-standing studies, osteoarthritis is regarded as a non-immune degenerative disease because of its less remarkable synovitis in comparison to rheumatoid arthritis.^13^ However, emerging evidence reveals that immune cells and associated cytokines play a key role in the pathophysiology of OA. It is believed that the joint is an entire organ, composed of synovial membrane with infiltration of immune cells and cartilage, which represents the pathological phenotypes caused by direct damage or changes in the microenvironment and, in turn, stimulates the synovial membrane as a feedback loop. Immune cells such as macrophages, T cells, and B cells are reported to be included in the immune microenvironment of OA and function as immune modulators of progression of the OA, mostly^14^(Fig. 2).

#### Innate immune response in OA aging microenvironment

Innate immune response in OA mainly consists of macrophages, mast cells, and dendritic cells, which respond to damage signals from injured cells.^15^ Among them, macrophages play a central role, existing as pro-inflammatory M1 macrophages secreting Tumor necrosis factor-α (TNF-α), interleukin IL-1, IL-6, and IL-12, and anti-inflammatory M2 macrophages, known as wound-healing macrophages. Macrophages in different OA microenvironment perform different functions depending on their M1/M2 phenotypes.^16^ In OA, DAMPs such as cartilage fragments, aggrecan, and fibronectin, released from damaged cartilage recruit and activate peripheral and local monocytes and macrophages.^16^ Subsequently, macrophages polarize into M1 in the synovium. Mouse models confirm that increased M1 macrophages exacerbate OA, while M2 macrophages alleviate symptoms.^17^ In 2018, Bai et al. developed OA models in mice with tuberous sclerosis complex 1 (TSC1) deletion specifically in the myeloid lineage, which could greatly increase M1 polarization. The results showed that the accumulation of M1 macrophages exacerbated experimental OA. Comparatively, Bai et al. found M2 macrophages significantly alleviated OA symptoms in mice via the construction of an OA model in mice with Ras homolog, mTORC1 binding (Rheb) deletion specifically in macrophages, which had been proved to increase M2 polarization.^17^ Recent single-cell studies also identified a distinct CX3C motif chemokine receptor 1 (CX3CR1)⁺ macrophage subset in the synovium with protective functions, highlighting the complexity and heterogeneity of synovial macrophages.^18^ Hence, defining the function of macrophage subclusters and the roles of macrophage polarization are key research priorities in OA aging microenvironment.

During the process of aging, there is more and more evidence suggesting that macrophage senescence plays a vital role in OA pathology. In 2023, Maerz et al. performed single-cell sequencing analysis of murine synovium from OA mice induced by Anterior cruciate ligament rupture (ACLR) surgery and from controls. Results showed that synovial macrophages in OA tend to perform as senescent phenotypes,^19^ indicated by increased expression of Cdkn1a (p21) in the OA group compared to the sham group, which is a typical marker of cellular senescence.^20^ Consistently, in 2023, Martinez-Barbera et al. proved that p16INK4a-expressing senescent macrophages expressed a subset of SASP, such as Bone morphogenetic protein 2 (Bmp2), IL-10, C-C motif ligand 2 (Ccl2), Ccl7, Ccl8, Ccl24, CXC motif chemokine ligand 13 (Cxcl13), and Ficolin A (Fcna), and also produced cytokines such as guanylate binding protein 2 (Gbp2) and interferon regulatory factor 1 (Irf1), indicated by single-cell sequencing of macrophages,^21^ which suggests senescent macrophages perform as the predominant pathologic cell types in the aging microenvironment. During aging and inflammatory response, M1-like macrophages, but not naive or M2 macrophages, accumulate in the tissue, implying potential relationships between senescent macrophages and polarization.^22^

Macrophage senescence is a driver of impaired efferocytosis of macrophages,^23^ which is termed a highly effective phagocytic removal of apoptotic cells performed by phagocytes, mainly tissue-resident macrophages.^24,25^ Subsequently, impaired efferocytosis is critical for the homeostatic imbalance and decreased tissue repair in a series of diseases, such as liver fibrosis, Non-alcoholic fatty liver disease (NASH), diabetic periodontitis, and so on,^26–28^ based on the loss of ability to augment proinflammatory cytokine production from apoptotic cells. Specifically, recent researchers set their sights on the correlation of efferocytosis and OA. In 2023, Appleton et al. found that OA is also correlated with impaired efferocytosis, indicated by accumulation of apoptotic cells.^29^ Consistently, in 2023, Zhang et al. proved that restoration of the phagocytic capacity of macrophages could reversely prevent obesity-associated OA,^30^ indicating modulation of efferocytosis as a promising approach for OA intervention. Besides, Knights et al. demonstrated that single cell sequencing of OA synovium revealed a subcluster of senescent macrophages which showed more expression of A disintegrin and metalloprotease 17(ADAM17), CD47, JAK and STAT3, indicating the efferocytosis of senescent macrophages in OA is impaired compared to other OA synovial macrophages.^19^ Mechanistically, IL-1 and TNF-α in the inflammatory microenvironment could induce ADAM17-dependent proteolytic cleavage of the macrophage phagocytic receptor, Triggering Receptor Expressed on Myeloid cells 2 (Trem2), which led to the increased pro-inflammatory microenvironment, indicating potential similar mechanisms in OA conditions in which IL-1 and TNF-α are two main proinflammatory cytokines.^28^ Previous studies implied strong correlations between macrophage senescence and efferocytosis in OA, which could be one of the typical features of synovial macrophages in the aging microenvironment of OA. Hence, focusing on the alteration of macrophage efferocytosis due to senescence might contribute to a better understanding of OA pathology. Based on these studies on the mechanisms of macrophage senescence, we should notice the unneglectable role of senescent macrophages in OA pathology.

Macrophage senescence is also a driving factor of decreased antigen presentation.^31^ Antigen presentation serves as a bond of innate immune response and adaptive immune response, in which antigens are digested and loaded onto major histocompatibility complex (MHC) class molecules of antigen-presenting cells (APC), further transported to another cell surface, which is essential for the exertion of cytotoxicity of T lymphocytes, and provision of help to cytokine production by B lymphocytes and plasma cell differentiation.^32,33^ Decreased antigen presentation caused by macrophage senescence contributes to subsequent affected adaptive immune response.

#### Adaptive immune response in OA aging microenvironment

The adaptive immune response in OA primarily involves B lymphocytes, T lymphocytes, and other immune cells. The dysregulation of adaptive immune responses significantly contributes to the progression of OA. Current research indicates that T lymphocytes play an important role in the pathogenesis of OA among these cells.

A series of numerical changes in T cells are found in the peripheral blood, synovial fluid, and synovial membranes of OA patients, indicating that T cells are also correlated with the pathogenesis of OA. For example, the synovial fluid and synovial membranes of OA show an increase in CD4^+^ T cell infiltration, with predominant T helper type 1 (Th1) cell polarization, which produces IL-2, interferon (IFN)-γ, TNF-α, lymphotoxins, and granulocyte-macrophage colony-stimulating factor to exacerbate OA progression.^34^ Also, CD4^+^ T cells could secrete macrophage inflammatory protein-1γ (MIP-1γ) and Nuclear factor kappa B (NF-κB) and induce MIP-1γ expression, leading to increased macrophage infiltration and osteoclast formation to promote OA progression.^35^ CD8^+^ T cells are found in the synovium of OA by flow cytometry and exacerbate OA by secreting Tissue Inhibitor of Metalloproteinases 1 (TIMP-1).^36^ Besides, compared to Rheumatoid arthritis (RA), Th1/17 cells were more frequent and enriched in inflammatory OA.^37^ Single cell sequencing analysis of OA synovium revealed various T cell subclusters, such as CCR7^+^ T cell, Regulatory T cells (Treg) cells, T peripheral helper (Tph) and T follicular helper (Tfh) cells, GZMK^+^ T cells, GNLY^+^ GZMB^+^ T cells, and GZMK^+^ GZMB^+^ T cells.^38^ Specific subsets of T cells have been proved involved in the pathogenesis of OA. Treg cells are one of the most concerned types of T cells in OA, which suppress the activation and proliferation of T cells, benefit for alleviation of inflammatory diseases and immune tolerance via immunomodulation and production of cytokines such as IL-10. Besides, the imbalance of Treg cells and T helper 17 cells (Th17) could significantly influence OA progression. Xia et al. reported that metallothionein 1 (MT1) knockout increased Th17 differentiation and inhibited Treg populations, which consequently led to exacerbated inflammation and cartilage erosion in OA. Single cell RNA sequencing indicated that compared with RA, the infiltration of CD4^+^ CD25^+^ Foxp3hi Treg cell cluster is much less in OA, which implied the important role of immuno-regulatory of Treg in inflammatory arthritis.^38^ As a result, series of studies focused on the regulation of Treg cells for treatment of OA and obtained satisfying efficacy. For example, Kim et al. reported that lipid nanoparticles for modulating Treg cells in an antigen-specific manner could greatly modulate cytokine secretion and inhibit infiltration of immune cells, further alleviated OA and reduced pain.^39^ Hence, biomaterial-based or drug-based approaches targeting T lymphocytes in OA have gradually been paid attention due to the critical effects of T cells in modulating OA microenvironment.

Nevertheless, the function of immune cells is another criterion for evaluating the condition, in addition to the quantity of T cells. For example, Treg cells are found to be elevated in the blood of OA patients, yet IL-10 secretion from Treg cells is diminished, indicating the declining function of Treg cells in OA.^40^ The number of other T cells, such as Th9 cells, has not been significantly changed in the synovial fluid or membrane. However, IL-9, produced by Th9 cells, has been detected increased in the peripheral blood and synovial fluid of OA patients.^41^ The reasons for these conditions might be triggered by the involvement of other cells since cell-cell interaction is also common in the pathology of OA. For example, Th2 cells and secreted IL-4 can drive polarization toward M2 macrophages, while IFN-γ secreted by CD4^+^ T cells induces M1 polarization. Both cytokines could be detected in the synovial fluid of OA.^35,42,43^

T cells are involved in a variety of inflammatory, malignant, or autoimmune diseases and have an important role to play in disease progression as well as therapy. Therefore, it is possible to use T cell-modified therapies, such as CD19-CAR (chimeric antigen receptors) T cell therapy in RA,^44^ as a possible treatment for remodeling the local pathological microenvironment. In OA, possible Car-T therapy could target biomarkers of inflammatory or senescent immune cells, such as CD9, CD40 and CD86 in synovial macrophages of OA which were found in single cell sequencing data of murine ACLR model.^19^ Modulation of the inflammation cascade exerted by Car-T therapy might be modulated via gene editing or combined targeting of Treg cells, benefit for both remodeling aging microenvironment and inhibiting Car-T treatment-mediated inflammatory response. Definitely, Car-T therapy calls for appropriate targets for therapeutic efficacy, which needs more investigations of mechanisms in cellular level. Moreover, nanomedicines targeting subsets of T cells, such as Treg cells,^45^ are generally also involved in alterations of the immune level, which is considered a potential therapeutic strategy for OA. However, it is important to note that they are still in the preliminary stage of exploration in the biomedical field. Therefore, the accurate regulation of T lymphocytes during the progression of OA is a necessary focus for future research.

As for B cells, current studies are not as abundant as for other immune cells, and the detection of B cells is difficult. In 1978, Pringle et al. first confirmed the existence of antibodies secreted by plasma cells in the synovium in OA by immunofluorescence. Besides, Shiokawa et al. confirmed the immunoglobulin transcripts of B cells in the synovial membranes of six patients with OA.^46,47^ It is believed that B lymphocyte (CD20^+^ in patients), especially plasma cells (CD138^+^ in patients), infiltration is associated with the degree of synovial inflammation.^46^ Thus, B cells could not be detected in the synovium of all OA patients (just half of OA cases, the same as the research studied by Revell et al.).^48–50^ Besides, to confirm the differentiation of B cells in OA synovium, it is found that no germinal center is seen in the local lesion, but B cells still have antigen selectivity (maybe antigenically activated before migration) and finally differentiate into plasma cells.^51^ Qi et al. reported that the critical regulatory factors for plasma differentiation include both TET2 and TET3, of which deletion in B cells caused reduced plasma cell and IgG secretion.^52^ We noticed that IgG has an obvious function of immuno-regulation, partly through the ability of opsonization. IgG has the Fcgamma domain which could be directly captured by FcR on macrophages or neutrophils which could enhance the phagocytosis of cells for bacteria, virus et al.^53^ As a result, IgG in joint produced by plasma cells potentially could induce the efferocytosis of synovial macrophages, which is benefit for OA remission. Due to the immuno-regulatory role of IgG towards virus infection and targeting antigen, it is to a great extent possible that IgG plays a vital role in combating inflammation in local joint. To be mentioned, recently Ma et al. demonstrated that IgG could induce a pro-senescent state in macrophages and microglia, thereby exacerbating tissue aging.^54^ Thus, the role of B cells, plasma cells and IgG is still confused due to inadequate investigation and needs further studies.

Since direct detection of B cells in the synovium of OA is hard to perform, interferes of B cells were carried out to investigate the function of B cells in OA. B-cell-specific conditional deletion of the tuberous sclerosis 1 gene (CD19-TSC1 transgenic mice) leads to more severe chondrocyte degradation and an accelerated OA phenotype.^55^ They function as a source of IL-10, contributing to OA pathogenesis, especially IgM^+^ CD27^+^ B cells in the synovial fluid, which could be driven by CXCL13.^56,57^ Despite the confirmed existence and gene modulation of B cells in OA progression, our understanding of the exact function of B cells in the peripheral blood and local joint cavity of OA remains inadequate. B cells have a series of subsets that play different roles in a variety of diseases. Only a thorough knowledge of the classification and functions of OA synovial B cells will allow for the study of association between adaptive immune response and OA pathology, as well as the targeting of immune cells except macrophages, in order to better control the immune homeostatic imbalance in OA.

Compared to rheumatoid arthritis (RA), OA is a more immunosuppressive disease, since the adaptive immune response is much less active in the OA course. Raychaudhuri et al. used single-cell RNA sequencing and flow cytometry to investigate T cells, B cells, monocytes, and fibroblasts from 36 samples of synovial tissue from RA patients and 15 samples of synovial tissue from OA patients. It was found that leukocyte-rich RA tissues had substantial infiltration of synovial T cells (CD45^+^ CD3^+^) and B cells (CD45^+^ CD3^−^ CD19^+^) which are significantly more than that in OA tissues, indicating less adaptive immune response in OA than in RA.^38^ Consistently, Zhang et al. also detected synovial cells in OA (*n* = 4) and RA patients (*n* = 15) via single-cell sequencing analysis. It was reported that the cell cluster correlated with immune regulations, such as regulation of B cell and T cell differentiation, significantly increased active RA compared to OA patients.^58^ The results suggested decreased antigen presentation in OA compared to RA which could lead to a decrease in B cell and T cell response. Accordingly, it is worthwhile to deeply investigate the function of macrophage antigen presentation affected by senescence in the OA microenvironment.^59^

#### Signaling pathways of immune response in OA aging microenvironment

Signaling molecules and pathways of immune responses and inflammation are involved in the pathogenesis of OA. Wnt signaling, NF-κB pathway, AMP-activated protein kinase (AMPK) pathway, mammalian target of rapamycin (mTOR) pathway, Hypoxia-inducible factors (HIFs) pathway, focal adhesion pathway, and fibroblast growth factor (FGF) signaling pathways are all correlated with the immune response in OA pathology.^60^ Besides, some studies showed that interfering with inflammatory molecules or pathways can cause an imbalance of immunological homeostasis. For example, inhibiting Neurogenic locus notch homolog protein 1 (Notch 1), T cell regulator), IL-1β (macrophage, T cells secreted), IL-6 (B cell regulator), and suppressing NF-κB signaling pathways (regulators of innate and adaptive immune responses) can attenuate aging-induced arthritis.^61–63^ Furthermore, the Notch 3 pathway has been linked to inflammation by modulating fibroblast identity.^64^ Also, chemokine axises such as CCL2/CCR2 (macrophage regulator) contribute to monocyte recruitment and OA symptoms.^65^

Although the aforementioned modulation of inflammation-correlated molecules and pathways can be used as an approach to target specific pro-inflammatory factors, immunomodulation via interfering with a single cytokine may be insufficient for improving the complex joint microenvironment, resulting in failure of OA control. To obtain better therapeutic efficacy to alleviate the immune homeostatic imbalance in OA, multiple cytokine-targeted strategies are gradually becoming a hot research topic in the immunomodulatory field of OA at present. From a critical point of view, the choice of targeted cytokines should be selected over others in a disease-specific pattern with clear rationale, not only to maximize therapeutic outcomes in different types or stages of OA but also to consider cost, scalability, and clinical translation. In addition, sustained-release systems targeting specific cytokines are expected to be used in different pathological conditions due to their unique properties, especially in water-soluble assemblies that show great potential in medical applications in OA joint cavities full of synovial fluid.

### Cellular senescence in aging microenvironment of OA

**Fig. 3:**
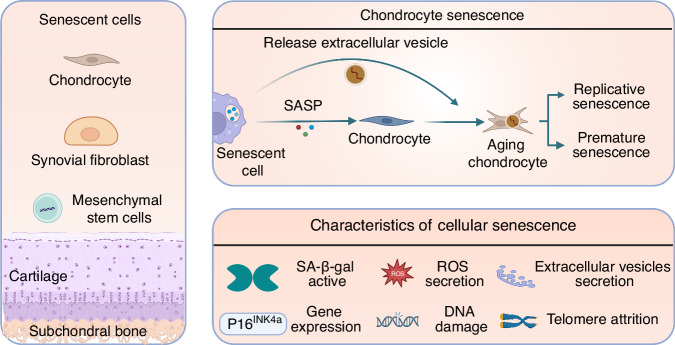
Cellular senescence in OA joints. Senescence in OA joints includes various cell types such as chondrocytes, fibroblasts, mesenchymal stem cells (MSCs), etc. The senescence of these cells accelerates joint aging through the secretion of cytokines or senescence-associated secretory phenotypes (SASPs) and the absorption of extracellular vesicles (EVS), leading to the progression of OA and joint aging

Cellular senescence is characterized by a series of molecular and functional hallmarks, including cell cycle arrest driven by the upregulation of cyclin-dependent kinase inhibitors such as p21 (CDKN1A) and p16 (CDKN2A), which are considered primary markers for senescence detection.^66^ Additional features include DNA damage (e.g., γ-H2AX foci at telomeres), nuclear envelope erosion (e.g., LMNB1 downregulation), senescence-associated secretory phenotype (SASP) involving pro-inflammatory cytokines like IL-6 and IL-8, and metabolic reprogramming.^66,67^ However, senescence markers exhibit significant tissue heterogeneity; for example, brain neurons and glial cells display distinct senescence profiles compared to liver or immune cells. Recent guidelines, such as the MICSE framework (Cell, 2024) and SenNet recommendations (Nature Reviews Molecular Cell Biology, 2024), emphasize multi-marker validation (e.g., combining p16/p21 with SASP factors or tissue-specific markers) to avoid false positives, as single markers like SA-β-gal or HMGB1 lack specificity.^66,67^ Advanced models, including conditional knockout mice and spatial omics technologies, are recommended to address context-dependent variability and improve in vivo detection accuracy. More and more researchers have realized that cellular senescence is an important pathogenesis in OA.^68,69^ Senescent cells (SNCs) in joints mainly include chondrocytes,^70–72^ synovial fibroblasts,^73^ cells in subchondral bone,^74^ such as mesenchymal stem cells, etc. In OA, cellular senescence is characterized by activation of senescence-associated beta-galactosidase (SA-β-gal), increased p16INK4a expression, telomere attrition, reactive oxygen species (ROS) secretion, activated DNA damage response, increased extracellular vesicle secretion, etc.^75–78^ However, due to the complexity of the senescent cells, more precise and accurate biomarkers are required to identify senescent cells in OA.^7^ It is believed that aging and cartilage injury (leading to abnormal mechanical loading) can accelerate chondrocyte senescence directly or indirectly, and DNA damage and oxidative stress could cause the senescent cells to accumulate in the OA joint.^7^ There are two basic pathological features associated with OA, which are loss of cartilage and alterations in subchondral bone architecture.^79^ Cellular senescence in the aging microenvironment of joints exhibits these two degenerative characteristics, which could further aggravate cartilage damage (Fig. 3).

On the one hand, senescent cells in the joint microenvironment lead to the progression of OA with limited extracellular matrix regeneration potential. Chondrocytes, as the major cell type in cartilage, are responsible for producing the extracellular matrix, which is the most abundant component of cartilage and accounts for the majority of its volume.^80^ Senescent chondrocytes are regarded to be the most involved cell type affecting OA through senescent phenotypes. For example, Swahn et al. performed single cell sequencing of articular cartilage and meniscus tissues from healthy (*n* = 6, *n* = 7) and OA (*n* = 6, *n* = 6) knees, and newly discovered a subcluster of chondrocytes which shows significant senescent phenotype, represented by enhanced expression of CDKN2A and increased expression of SASPs, termed as pathogenic subset of chondrocytes.^9^ The result visually presented senescent chondrocytes which might secret SASPs to exacerbate OA via single cell sequencing. Also, senescent chondrocytes display restricted extracellular matrix regeneration potential, which is featured by telomere shortening and increased senescence-associated β-galactosidase (SA-β-gal) activity.^81,82^ Senescence of chondrocytes may be triggered by replicative senescence (through the p53/p21 pathway) or stress-induced premature senescence (through chondrocyte apoptosis and the p38/p16 pathway). Interestingly, Jeon et al. revealed that aging chondrocytes can secrete a greater number of extracellular vesicles (EVs), which can be absorbed by normal cells, trigger senescence in adjacent healthy cells in the aging microenvironment via modulation of microRNA, and, ultimately, exacerbate OA.^83^ The results indicated that despite SASP, the secreted cellular components from senescent cells also serve as drivers of aging. Besides, senescent synovial fibroblasts may also possibly contribute to OA pathophysiology, as the expression of p16 is prevalent in OA synovial tissue samples. Senescent synovial cells tend to exhibit an inflammatory phenotype, secreting IL-6, CXCL-8, Matrix metalloproteinase-3 (MMP-3), etc., and expressing collagen type I with the Wnt signaling pathway activating in OA.^73,84^ In turn, OA could be induced when senescent fibroblasts are transplanted into the knee joint.^85^ Elisseeff et al. found that senescent synovial fibroblasts can steer naive T cells toward Th17 or Th1, and in return, Th17 can cause senescence in OA fibroblasts, which indicates immunologically influencing senescent cells to treat OA may be feasible.^86^ However, some other studies hold alternative perspectives. They think p16INK4A and SA-β-gal positive cells found in synovium may be a phenotype of polarized macrophages induced by OA, since synovial cells and macrophages are difficult to distinguish, and p16 and SA-β-gal positive macrophages can be triggered by injection of SNCs.^83^ Thus, further research on accurate senescent cell types is required.

Senescent cells produce SASPs to accelerate the progression of OA. Senescent chondrocytes can secrete chemokines [CCL2, CCL4, Growth-regulated oncogene α (GROα)], cytokines [IL-1, IL-6, IL-7, IL-8, oncostatin M (OSM), Granulocyte-macrophage colony-stimulating factor (GM-CSF), TNF-α], proteases (MMP1, MMP3, MMP10, MMP13, ADAMTS5, ADAMSTS7), and growth factors [TGF-β, Insulin-like growth factor-binding protein (IGFBP), Vascular Endothelial Growth Factor (VEGF)] that alter the microenvironment of cartilage and may also degrade the structure of the extracellular matrix directly, leading to OA.^7,87,88^ SASPs released by senescent cells in the aging microenvironment of OA contribute to the development and aggravation of OA and cause pain in OA. Multiple signaling pathways, such as the DNA damage response, p38 mitogen-activated protein kinase (MAPK), and cyclic GMP-AMP synthase (cGAS)/ stimulator of interferon gene (STING) pathways, have been linked with SASP regulation through the activation of downstream NF-κB and CCAAT/enhancer-binding protein beta (C/EBPβ).^83^ To be mentioned, SASP could not only drive the degradation of the extracellular matrix of cartilage but can also impact the autocrine of each other. For example, TNF-α could upregulate the expression of MMPs and ADAMTS, while IL-1β could significantly increase the expression of MMPs in human chondrocytes, which is to say, senescence and secretion of SASP might be in a vicious cycle.^89^ Hence, designing targeted therapies against SASPs in synovial fluid of OA is a more direct approach as potential interventions, yet current therapies showed low efficacy in combined targeting of different SASPs, suggesting more accurate and accessible modulations of senescent-related cytokines in the local joint of OA need to be explored.

### Stem cell exhaustion in aging microenvironment of OA

In vivo, native mesenchymal stem cells are abundant in joints, called joint-resident MSCs, which exist in the subchondral bone, cartilage, synovial fluid, synovium, and adipose tissue, meniscus, ligament, and fat pad, and are crucial for chondrogenesis and cartilage repair.^90,91^ In early-stage OA injuries, especially superficial injuries of cartilage, synovial fluid-resident and synovium-resident MSCs could be recruited to the injury site, adhering to cartilage in a favorable biochemical and biomechanical environment, initiated by the soluble factors released after injury,^92^ resulting in a numerical increase of MSCs in the lesion. It has been reviewed that, despite the direct ability of chondrogenesis and osteogenesis of MSCs to alleviate OA, MSCs also perform a series of functions of repair and regeneration through other indirect pathways, such as reduction of inflammation by decreasing proliferation and infiltration of immune cells, reduction of apoptosis of functional cells, and induction of angiogenesis to enhance the blood supply in local lesion.^93^ Besides, MSCs could secrete cytokines to regulate the OA microenvironment, including BMP-2 and insulin-like growth factor-1 (IGF-1), which enhance cellular regeneration while decreasing OA inflammatory and immune reactions.^93^ Furthermore, MSC-secreted exosomes could regulate matrix expression to reduce joint inflammation and promote regeneration.^94,95^

However, in the aging microenvironment of OA, the function of MSCs declines, which indicates stem cell exhaustion to a certain extent.^96^ Evidence demonstrated that dysfunction and loss of proliferation ability in both skeletal muscle- and bone-derived MSCs were detected in OA patients compared with healthy donors, indicating stem cell exhaustion in OA.^97^ Also, when comparing primary OA and dysplastic OA, researchers found that subchondral bone-derived MSCs showed deficient osteogenic and chondrogenic properties.^98^ As age goes on, the chondrogenic activity of the periosteal MSCs decreases compared to donors under 30 years old,^99^ indicating that both aging of joints and OA might result from stem cell exhaustion. Stem cell exhaustion in osteoarthritis shows both function deficiency and number loss of joint local MSCs. As we have mentioned above, MSCs demonstrated deficient osteogenic and chondrogenic properties in OA,^98^ while the migration ability of MSCs is also altered in osteoarthritis. As well-known, the migration of MSCs is crucial in the processes of chondrogenesis and cartilage repairment, as proved by previous experiments.^96^ It was also found that MSC migration is sensitive to inflammatory factors like Stromal Cell-Derived Factor 1 (SDF-1) and IL-8 in the presence of lamin A, a molecule usually occurring in OA, which is different from normal MSCs. As for the numerical loss of MSCs, it was found that MSCs from primary OA show lower gene expression of the leptin receptor, which is one of the MSC markers, indicating that the pathology of primary OA is accompanied by bone MSC exhaustion.^100^ Thus, function deficiency and number loss can be identified as features of stem cell exhaustion in OA.

Stem cell senescence is also a typical feature of stem cell exhaustion because senescence of stem cells leads to functional decline,^101^ which was observed in OA progression. Firstly, the senescence of MSCs in subchondral bone was identified, and the seno-suppressive paracrine effect was proven to be attenuated in OA, along with the loss of the ability to self-renew and regenerate chondrocytes.^102^ Malaise et al. found that the p16INK4a positive-senescent MSCs can also trigger the OA phenotype when injected into 2-month-old mice.^102^ Conversely, rejuvenating aged MSCs by overexpressing Yes-associated protein (YAP) or Forkhead box D1 (FOXD1) could attenuate the development of osteoarthritis in mice.^103^ Furthermore, skeletal stem cells (SSCs) in subchondral bone decreased significantly with age, and senescent SSCs showed diminished chondrogenesis, which may contribute to OA.^104^ Within the context of OA, there are distinct roles and differences between stem cell senescence and stem cell exhaustion. Stem cell senescence refers to the state where stem cells undergo functional decline due to aging or other stress factors. It is characterized by reduced proliferative capacity, altered differentiation potential, and changes in secretory functions. For instance, studies on induced pluripotent stem cells (iPSCs) have shown that senescent iPSCs exhibit impaired differentiation capabilities and increased secretion of pro-inflammatory factors, which can negatively impact tissue repair and regeneration. Senescent stem cells can also spread their senescent phenotype to neighboring cells through paracrine signaling, thereby exacerbating the aging of the tissue microenvironment.^105^ Stem cell exhaustion encompasses both the functional decline and numerical reduction of stem cells. It is a broader concept that includes the loss of stem cell quality and quantity in the tissue microenvironment. This can result from multiple factors such as aging, mechanical stress, and the influence of other pathological features like senescent cells and immune homeostatic imbalance. For example, research has demonstrated that with age, the number and function of bone marrow mesenchymal stem cells (BMSCs) decline. The self-renewal and differentiation abilities of these cells are weakened, leading to a reduced capacity for tissue repair and regeneration.^106^ In summary, stem cell senescence is a specific manifestation of stem cell exhaustion, directly reflecting the functional decline of stem cells. Stem cell exhaustion, on the other hand, is a more comprehensive term that includes both the functional decline and numerical reduction of stem cells. In the pathogenesis of OA, both stem cell senescence and exhaustion play interrelated roles, collectively impacting the repair and regeneration capacity of joint tissues.

**Fig. 4:**
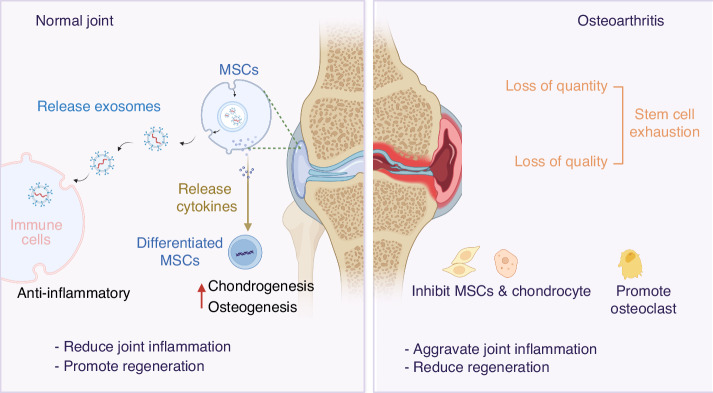
Stem cell exhaustion in the aging microenvironment. In early-stage of OA injury, MSCs can be recruited to the site of injury due to inflammatory factors released after injury, which perform a range of repair and regenerative functions through other indirect pathways, such as anti-inflammation by reducing the proliferation and infiltration of immune cells. However, in long-term chronic inflammation of OA, stem cells tend to form as exhausted status, represented by loss of quality and quantity, which are harder to reduce inflammation and differentiate

Notably, the interaction between other pathological features and stem cell exhaustion contributes to the OA progression. Exhausted MSCs showed insufficient intrinsic and seno-suppressive paracrine capabilities, which further aggravated the cellular senescence of chondrocytes and osteoarthritis.^102^ Another powerful evidence of this interaction is that MSCs can suppress inflammation through multiple mechanisms, such as exosomes, and this kind of suppression can promote chondrogenesis.^94,107^ In summary, stem cell exhaustion is a crucial pathogenesis mechanism in age-related osteoarthritis (Fig. 4).

### Interaction between key factors in the aging microenvironment of OA

Importantly, the three key factors in the aging microenvironment of OA, including immune homeostatic imbalance, cellular senescence, and stem cell exhaustion, interact with each other to form this complex pathological environment. Here, we mainly review the potential correlation and shared signaling pathways between the three key factors of aging microenvironment.

The interaction between immune cells and stem cells has proven vital for regeneration in a wide range of tissues. In early stage of OA, stem cells, such as articular cartilage stem cells and synovial membrane mesenchymal stem cells, are activated as an initial attempt for self-repair.^108,109^ However, OA immune homeostatic imbalance gradually results in stem cell exhaustion, in which OA synovial macrophages secrets proinflammatory factors such as IL-1, IL-6, IL-12, and TNF-α.^110^ Tong et al. demonstrated that IL-1β potently impaired the responsiveness of articular cartilage stem cells, which could be rescued via NF-κB pathway inhibitor.^109^ Another study also claimed the irreversible influence of inflammatory challenges on stem cell exhaustion. Bogeska et al. found that intervention of polycytidylic acid, an ideal drug for inflammatory challenges, greatly decreased the clonogenic potential of stem cells, which, importantly, is irreversible, as evidenced by a 12-month follow-up of mice after sterile inflammatory challenge.^111^ These results implied that the interaction of immune response and stem cells could contribute to OA progressive cartilage degeneration, indicating that early intervention of immune homeostatic imbalance is the window of opportunity for OA treatment. SASPs secreted by senescent cells such as IL-1 and TNF-α, are the main sources for triggering senescence-related pathways in stem cells, including PI3K/AKT/mTOR signaling.^112^ Interestingly, stem cell exhaustion contributes to the induction of SASPs and consequently OA progression, which could form a negative loop in the OA aging microenvironment. Chondrogenic progenitor cells are gradually paid attention in OA pathogenesis these years, specifically marked by PRG4 (lubricin) compared with MSCs. Justin et al. demonstrated that chondrogenic progenitor cells exhibit senescent phenotype with functional decline in OA, which led to increased secretion of ROS, IL-6 and IL-8 to exacerbate the course.^113^ Besides, Gnani et al. proved that aged stem cells could secrete SASPs, limiting the proliferation rate of neighboring cells and triggering activation of a pro-inflammatory program,^114^ implying the likely mechanisms by which stem cell exhaustion exacerbated immune homeostatic imbalance in OA.

Cellular senescence, including chondrocyte and synoviocyte senescence as reported, to a great extent accelerates immune homeostatic imbalance and stem cell exhaustion.^83^ Deng et al. demonstrated that SASPs such as IL-6 contributed to enhanced expression of CD73 in macrophages via the JAK-STAT3 signaling pathway, positively correlated with senescent marker, CDKN1A expression, which could consequently lead to T cell dysfunction and immunosuppression according to single-cell analysis.^115^ IL-6 is one of the typical SASPs secreted by OA macrophages which could be inhibited via dasatinib plus quercetin, the senolytic cocktail proved effective against OA.^116,117^ In OA, Faust et al. reported that senescent cells could skew naive T cells toward Th17 to secrete IL-17 via Wnt signaling, which, in turn, reduced CD4^+^, IL4^+^ anti-inflammatory T cells and caused increased senescent cells.^86^ This represents an interaction between cellular senescence and immune homeostatic imbalance in OA. As for stem cell exhaustion, aging-associated changes include defects in maintenance of stem cell quiescence, differentiation ability and bias, and clonal expansion, which results from increased senescent cells and SASPs in the niche.^118^ For example, Dulken et al. demonstrated that single-cell RNA sequencing of young and old niches in mice revealed that senescent cell-derived interferon-γ (IFN-γ), one of OA-related SASPs, is responsible for the decreased proliferation of stem cells.^119^ Another study reported by Yousef et al. proved that single-cell sequencing of young and aged epithelial cells showed different expression of vascular cell adhesion molecule 1 (VCAM1), which was found in aged serum and consequently decreased the proliferative ability of progenitor cells.^120^ Notably, VCAM1 has been proved as a strong and independent predictor of severe OA and biological target for human aging.^121,122^ The above results showed that, stem cell exhaustion is, undoubtedly, susceptible to cellular senescence in the local microenvironment.

**Fig. 5:**
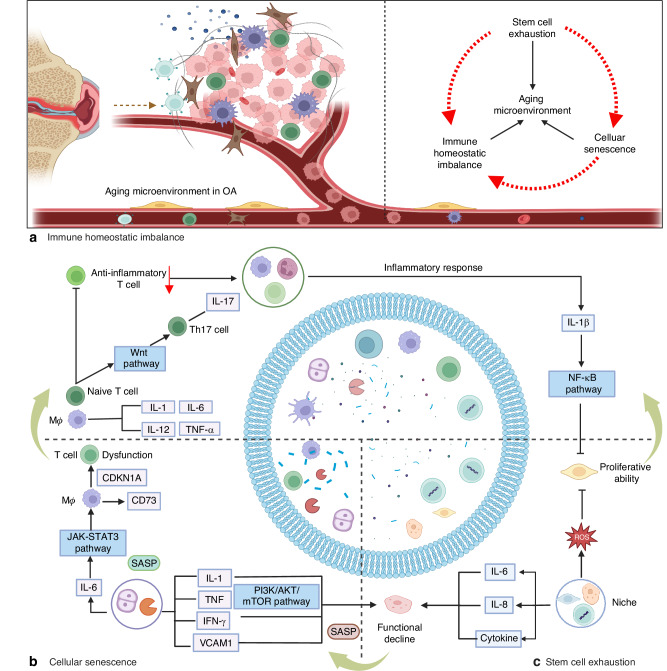
The interaction of key factors in the aging microenvironment of OA. Inflammatory factors affect stem cell functions, while stem cell exhaustion can in turn disrupt immune balance via ROS and cytokines. Cellular senescence leads to immune homeostatic imbalance and stem cell exhaustion via SASPs and specific signaling pathways. Interestingly, stem cell exhaustion contributes to the induction of SASPs

To conclude, immune homeostatic imbalance, cellular senescence, and stem cell exhaustion are closely correlated and interact with each other (Fig. 5). A deeper acknowledgement of the relationship could contribute to reveal more important pathological factors, which could be of significant benefit for clinically targeted therapies.

### Heterogeneity of OA aging microenvironment

We have reviewed that the aging microenvironment of OA is commonly composed of immune homeostatic imbalance, cellular senescence, and stem cell exhaustion. However, there is still heterogeneity of these three components between different patients and disease courses. Along with the development of single cell RNA sequencing and spatial transcriptomics, acknowledgement of heterogeneity of the aging microenvironment of OA will be beneficial for precise therapeutic strategies according to patient-specific and stage-specific properties.

Boer et al. reported a study on *Cell* in 2021, emphasizing the importance of acknowledging the heterogeneity of OA, in which they conducted a genome-wide association study meta-analysis across 826 690 individuals (177 517 with OA) and summarized similarities and differences of genetic risk according to gender, onset and joint sites of OA. We noticed that the team identified three new female-specific osteoarthritis risk variants, among which FANCL, causative for premature ovary insufficiency, showed significance in the female-only total hip replacement phenotype, implying the heterogeneity of OA senescence due to gender. Via comparison of association signals across OA phenotypes, most of the single nucleotide variants (SNVs) associated with knee, hip, and knee and/or hip OA have a larger effect size on the respective joint replacement-defined phenotypes. rs28929474 (missense variant in SERPINA1), thought to improve inflammation, showed a stronger association with total hip replacement (THR), total knee replacement (TKR), and total knee/hip replacement compared with hip or knee OA.^123^ This multicohort genome-wide association meta-analysis of OA points toward potential targets for therapeutic intervention.

Single-cell sequencing showed great efficacy in the investigation of the heterogeneity of OA due to accurate analysis of subclusters. For example, Nanus et al. reported that PCA and t-SNE analysis revealed seven distinct subsets of synovial cells between early- and end-stage knee OA and between painful and non-painful synovial sites. They found CXCL3, PTGDS, DNAJB1 and HTRA are more expressed in late-stage OA, in which CXCL3 is a typical factor secreted by inflammatory cells, indicating the immune homeostatic imbalance aggravates along with disease progression. By contrast, clusters dominant in early-stage OA showed functions for mediating cellular migration, the development of neurons, and early inflammation.^124^ These results indicated the heterogeneity of immune imbalance in different onsets of OA. Besides, Komaravolu et al. demonstrated via single- cell sequencing analysis that pro-fibrotic genes increased while pro-inflammatory genes such as Adam17, Cd14, Icam1, Csf1r, and Casp1 decreased in OA joints of female mice, but not male mice.^125^ These results stress the heterogeneity of immune imbalance due to gender or disease courses via single-cell analysis. In addition, the identification of novel clusters in OA has been significantly facilitated by the integration of multi-omics data, which includes single-cell and spatial transcriptomics analysis. Fan et al. unveiled a pre-inflammatory chondrocyte population (preInfC, marked by IFI16 and IFI27) and an inflammatory chondrocyte population (InfC, marked by CXCL8, CD74 and GPR183) in OA via spatial transcriptomics analysis. The two novel clusters they demonstrated are responsible for focal adhesion, regulation of angiogenesis (preInfC) and inflammatory response and innate immune response (InfC), in which InfC was shown to be uniquely identified in OA patients compared to non-OA patients.^126^ This study not only newly found subclusters of chondrocytes in OA but also revealed their inflammatory signature via single-cell spatial transcriptomics. We believe that deeper investigations would be performed via multi-omics analysis for uncovering the heterogeneity of the OA aging microenvironment. The integration of single-cell sequencing with multi-omics analysis provides unprecedented insights into the heterogeneity of the aging microenvironment and the complex pathogenesis of OA. The discovery of novel subclusters and targeting gender-specific, phenotype-specific, and stage-specific molecules within the aging microenvironment of OA offer critical theoretical foundations and technical support for the development of personalized therapies and drug development.

## Strategy on regulating aging microenvironment in OA

It is widely recognized that the usual treatment approach involves topical or oral nonsteroidal anti-inflammatory drugs (NSAIDs), as well as intraarticular glucocorticoid injections, steroid injections, and chondroitin sulfate. These treatment options were recommended in the 2019 guideline for managing OA.^127^ Additionally, traditional methods of reducing inflammation involve using Cyclooxygenase 2 (COX2) inhibitors, proton pump inhibitors (PPIs), misoprostol, and salicylate substitutes.^128^ Nevertheless, these therapies have certain limitations in their application in clinical practice. The development of OA is significantly influenced by the core components of the aging microenvironment, including immune homeostatic imbalance, senescent cells, and stem cell exhaustion. It is important to highlight that the pathological changes of the microenvironment precede the degeneration of functional cells. Exploring potential therapies that target the regulation of the aging microenvironment could be a significant direction of research for preventing or alleviating osteoarthritis.

### Regulating immune homeostatic imbalance to prevent and treat osteoarthritis

Regulation of the immune system in OA has been gradually focused on the field of early intervention of OA because immune homeostatic imbalance occurs partly before OA symptoms and influences OA progression. It is noteworthy that the immune cells, inflammatory factors or cytokines, and inflammatory pathways could serve as targets via the delivery of drugs or biomaterials. As listed in Table 1, different strategies targeting the components of the immune system in OA joints, such as macrophages, IL-1, and TNF-α, were proven as valuable interventions in treating OA. The current immunomodulatory strategies for OA primarily target two key pathological components: macrophage polarization and inflammatory cytokine signaling. For macrophage-directed therapies, the principal mechanisms involve modulating macrophage polarization states and functional reprogramming to attenuate their pro-inflammatory activity. In contrast, cytokine-targeted interventions focus on suppressing the production and activity of multiple inflammatory mediators that drive OA progression.

**Table 1:** Strategies for modulating the OA aging microenvironment

Targets		Treatments	Mechanisms	Ref.
Immune homeostatic imbalance	Macrophages targeted	AP20187	Reduced osteophyte formation by controlling macrophage	^129^
		Kinsenoside	Repolarizing macrophages through inactivating NF-κB/MAPK signaling	^130^
		Quercetin, SiPGAM5 nanoparticles, Transplantation of Human Adipose-Derived Regenerative Cells	Polarizing M2 macrophages to alleviate OA	^110,135,136^
		TRPV1	Inhibiting M1 macrophage polarization via Ca^2+^/CaMKII/Nrf2 signaling pathway	^132^
		Fargesin	macrophage reprogramming by downregulating MAPK and NF-κB pathways	^133^
		Eucommia ulmoides Polysaccharides	Regulating the function of macrophage and alleviating OA	^134^
		Modified ZIF-8 nanoparticles	Reprogramming the Metabolic Pathway of Synovial Macrophages	^135^
		BMSC-derived exosomes	Regulating synovial macrophage polarization	^137^
Cellular senescence	Senolytics	Rapamycin	Inhibits mTORC1 and senescent cells	^138,139^
		UBX0101	Induces apoptosis of senescent chondrocytes	^143,144^
		Spermidine	Enhances autophagy of senescent chondrocytes	^147^
		Fenofibrate	Induces apoptosis and autophagy of senescent chondrocytes	^148^
		Ganciclovir	Targets p16INK4a-expressing cells	^143^
		ABT263	Enhances apoptosis of senescent cells and reduces inflammatory factors	^146^
		AT-406	Eliminates senescent chondrocytes	^145^
		Dasatinib and quercetin	Eliminates of senescent chondrogenic progenitor cells	^149^
	Senomorphics	MiR140	Retards the chondrocyte senescence	^151^
		embryonic stem cells	Inhibits mTORC1 and senescent cells	^152^
Stem cell exhaustion	Supplementation of stem cells	pluripotent stem cells	Induces apoptosis of senescent chondrocytes	^154^
		bone marrow-derived stem cells	Enhances autophagy of senescent chondrocytes	^155^
		adipose-derived stem cells	Induces apoptosis and autophagy of senescent chondrocytes	^156^
		synovium-derived stem cells	Targets p16INK4a-expressing cells	^157^
	Rejuvenation of stem cells	kartogenin	Regulation of transcription induces chondrogenesis	^161^
		peptide-hydrogel	Recruit mesenchymal stem cells and enhance chondrogenesis	^162^

The most direct way of maintaining a local immune balance of joints is interfering with immune cells mentioned above, which draws a lot of attention but is not practical because the regulation of immune cells needs to be improved. As for the most common occurrence of macrophages in OA, studies have focused on the direct depletion of macrophages hoping to cure OA. Macrophage depletion by a small molecule (AP20187) in Fas-induced apoptosis (MaFIA)-transgenic mice exactly reduced osteophyte formation but increased synovitis, which enhances OA,^129^ indicating that controlling macrophage polarization or targeting subpopulations of macrophages rather than deleting the whole macrophages may be more effective. In recent years, accumulating drugs have been found to either repolarize M1 macrophages to M2 macrophages or directly target the reduction of M1 macrophages, including kinsenoside, quercetin, capsaicin, fargesin, and eucommia ulmoides polysaccharides.^130–134^ Furthermore, bioengineering-combined therapy targeting M1 and M2 macrophages showed effectiveness, such as siPGAM5 nanoparticles (NPs), modified zeolitic imidazolate framework-8 (ZIF-8) NPs, and injection of adipose-derived regenerative cells (ADRCs).^110,135,136^ Despite the usage of drugs and biomaterials, extracellular vesicles have also been paid attention in the immunomodulation of OA. Zhang et al. investigated the functions of extracellular vesicles from bone marrow mesenchymal stem cells in OA. Results indicated that bone marrow mesenchymal stem cell-derived exosomes could delay the progression of osteoarthritis by inhibiting M1 macrophage production and promoting M2 macrophage generation,^137^ which suggested the potential usage of exosome-based intervention in modulating the immune system in OA. Till now, most research has focused on the regulation of synovial macrophages, yet because of the unclear pathological mechanisms of T lymphocytes and B lymphocytes in OA, there are few investigations of interventions targeting adaptive immune response in OA.

**Fig. 6:**
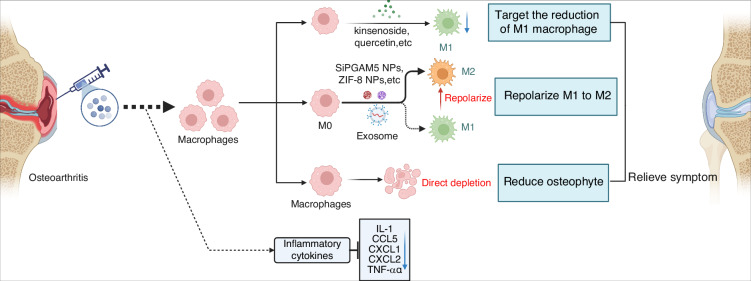
Intra-articular injection of agents targeting macrophages and macrophage-secreted inflammatory cytokines is an effective way to prevent OA. Mechanistically, current strategies focus on reprogramming of M1-to-M2 repolarization and reduction of inflammatory cytokines via soluble factors or gene editing via intraarticular injection

In OA treatment, IL-1 could be inhibited by rapamycin, which results in the decrease of SASP in human fibroblasts and autophagy activation, regulated by mTOR,^138,139^ meanwhile, Diacerein, AMG108, and Anakinra show inhibition of IL-1 as well.^140^ However, inhibiting IL-1 specifically shows limited therapeutic effects in OA. In OA treatment, strategies also focus on the regulation of inflammatory cytokine TNF-α, including the usage of Adalimumab, DLX105, and the C-terminal domain of PTHrP, which, however, has not been proven clinically.^140,141^ Due to the limited therapeutic efficacy of simply targeting TNF-α, it has been reported that multi-targeted proteins such as tristetraprolin could be dephosphorylated and activated to negatively regulate cytokines like TNF-α, CCL5, CXCL1, and CXCL2.^142^ These researches shed light on the modulation of immune homeostatic imbalance via directly targeting cytokines. Compared to cell-targeted therapies, the interference of OA cytokines might provide a more acceptable approach for patients in the clinic, yet the accurate targets still need to be validated (Fig. 6).

### Targeting senescent cells

Targeting senescent cells has non-ignorable effects in OA treatment. Up to now, to regulate the pathological aging microenvironment in OA through targeting senescent cells, the strategies can be mainly divided into two aspects: clearance or inhibition. Hence, it is still necessary to conduct basic research to gain insight into the aging microenvironment of OA at the genetic, molecular, and cellular levels. Notably, as listed in Table 1, the usage of senolytics and senomorphics has garnered considerable attention in recent years as a crucial aspect of modulating cellular senescence in OA. Current therapeutic strategies targeting cellular senescence in osteoarthritis employ two distinct pharmacological approaches with complementary mechanisms: Senolytics alleviate osteoarthritis progression by selectively inducing apoptosis or enhancing autophagy in senescent cells, while senomorphics exert therapeutic effects by modulating the SASP or decelerating cellular senescence progression.

#### Clearance of senescent cells in OA

In OA, eliminating senescent cells is termed a new therapeutic target as senolytics.^143^ The most common target of senolytics is modulating the apoptosis of senescent cells. As an example, early in 2017, Elisseeff et al. confirmed that the usage of senolytics in decreasing senescent cells in OA, which is represented by senolytic UBX0101, resulting in attenuation of post-traumatic OA and the formation of a prochondrogenic environment by inducing apoptosis of senescent cells and improving the cartilage-forming ability of chondrocytes.^143,144^ Besides, Yi et al. demonstrated that AT-406, which inhibits apoptosis inhibitor protein, could eliminate senescent chondrocytes to attenuate the development of osteoarthritis.^145^ What’s more, Liu et al. used ABT263 (navitoclax) to enhance apoptosis of senescent cells and reduce inflammatory factors in OA chondrocytes by injection both in vivo and in vitro, presenting a new targeting strategy for clinical use.^146^ Despite directly modulating apoptosis, some investigators also pay attention to regulating cell metabolisms to control senescence. As an example, Edwards et al. demonstrated spermidine as a type of senolytics in treating OA. What’s different is that they found that spermidine eliminated senescent cells via enhancing chondrocyte autophagy, which is another approach to modulate senescence rather than directly destroy senescent cells.^147^ Consistently, Caramés et al. also found fibrates (fenofibrate) as an agonist of autophagic flux, resulting in a reduction of senescent cells to protect against OA, which has been FDA-approved.^148^ Although the regulations of apoptosis and autophagy of cells in eliminating senescence showed efficacy, researchers are not satisfied with the targeted ability of drugs, leading to more accurate approaches to the elimination of senescence in OA. As an example, Elisseeff et al. demonstrated that the eliminating of p16INK4a-expressing cells via the use of ganciclovir greatly alleviated posttraumatic OA. Notably, they for the first time revealed the role of p16INK4a-expressing cells in OA via the construction of p16-3MR transgenic mice, which harbors a p16INK4a(Cdkn2a) promoter driving the expression of a fusion protein containing synthetic Renilla luciferase for better follow-up and removal of senescent cells in OA. This work to a great extent reminded researchers to pay attention to senescent cells that are specifically marked by senescent biomarkers, which guided future explorations in modulating cellular senescence in OA.^143^ Except for targeting senescent cells, the modulation of SASPs could also show therapeutic effects in eliminating cellular senescence in OA. For example, Gui et al. used dasatinib and quercetin (DQ) to decrease SASPs in the joint microenvironment of OA and promote cartilage regeneration via the elimination of senescent chondrogenic progenitor cells.^149^ Undoubtedly, in the aging microenvironment of OA, modulations of senescence mostly target chondrocytes; however, some researchers threw out a view that interfering with cellular senescence of synovial cells can also show an anti-arthritis effect.^150^ Unsatisfactorily, fewer researchers directly eliminated senescent immune cells in OA due to a lack of acknowledgment of related mechanisms, suggesting the need to treat OA senescence in a broader aspect.

#### Inhibition of senescent cells in OA

Inhibition of cellular senescence is another feasible way to prevent OA. Not only the elimination of senescent cells is effective in treating OA, but the retardation of senescent cells could attenuate the progression of early-stage OA as well. Researchers have found that intra-articularly injected miR140 could quickly induce molecular changes by inhibiting the expression of SA-β-Gal, p16INK4a, p21, p53, and H2A histone family member X (γ-H2AX), which retards the chondrocyte senescence to gain a noticeable alleviation of OA.^151^ Also, reduction of senescent cell accumulation shows effectiveness for treating OA as well. As an example, Zhou et al. found that parathyroid hormone treatment could inhibit phosphorylation of Smad3 and further inhibit the combination of the p16INK4a gene promoter region, resulting in ameliorating the degenerative changes of OA by reducing senescent cell accumulation and activating bone remodeling.^152^ To conclude, current strategies could not precisely inhibit specific clusters of senescent cells of OA in vivo clinically, mainly due to the untargeted risks and disapproval of ethics, while senomorphics, which could inhibit the adverse effects of senescent cells gently, also provide potential approaches to treat OA senescence. Importantly, to better target OA senescence, the complete atlas of the aging microenvironment of OA urgently needs to be identified, which is to a great extent beneficial for clinical interventions.

### Stem cell therapies

More and more OA patients have received stem cell therapy because of the multi-lineage differential potential, immunosuppressive, and self-renewal capabilities of stem cells, which could impressively improve pain and joint function.^93^ Since stem cell exhaustion is characterized by a change in the quantity and quality of stem cells, the treatment aimed at stem cells can mainly be divided into two aspects: supplementation of stem cells and rejuvenation of stem cells.

#### Supplementation of stem cells to treat OA

In OA, supplementation of stem cells refers mostly to transplantation of stem cells. It has been found that MSCs possess immunosuppressive properties and are deemed safe for clinical use by the FDA in the United States.^153^ Therapies based on the injection or transplantation of stem cells, including embryonic stem cells (ESCs) or induced pluripotent stem cells (iPSCs)-derived MSCs, bone marrow-derived stem cells (BMSCs), adipose-derived stem cells (ADSCs), umbilical cord blood-derived MSCs (UCB-MSCs) and synovium-derived stem cells (SMSCs) or stem cell-derived exosomes have been widely researched, in which BMSCs, ADSCs and UCB-MSCs have been proved effective to attenuate OA in clinic.^154–158^ As mentioned, a series of MSC therapies have been tested in randomized control trials (RCTs). MSCs have also demonstrated chondroprotective or regenerative effects on cartilage in 18/21 clinical studies, including 11/15 RCTs.^159^ Though various clinical trials have confirmed the effects of MSC supplements in OA treatment, the selection of basally fit MSC products involves selecting MSCs from a host of donors that are relevant in the context of OA, including immunomodulatory properties, angiogenic properties, and tissue remodeling properties. Specifically, not only supplements of MSCs from local joint microenvironments could benefit OA, but stem cells from other systems gain consistent effects. As an example, Wang et al. extracted exosomes from antler stem cells for OA treatment. Results showed that OA was greatly decreased via modulating the senescence of MSCs.^160^Also, the study reminds us of the importance of the maintenance of stemness in cell-based therapies of OA treatment. The supplementation of MSCs has been widely investigated and obtained decent therapeutic efficacy, yet in clinical usage, it is still hard to determine the amount and timepoint of MSC therapy. Consequently, more clinical trials with different groups of disease progression and individual features still need to be accomplished for better control of accurate injection of MSCs in OA intervention.

#### Rejuvenation of stem cells to treat OA

Because stem cell therapy has several restrictions, such as an uncertain long-term survival rate of MSCs and post-transplantation homing of MSCs,^161^ stem cell rejuvenation has been introduced as a new concept in recent years. In OA, rejuvenation of stem cells could be defined as regaining or increasing the ability of chondrogenesis of mesenchymal stem cells, which also shows effectiveness. Schultz et al. used an image-based high-throughput screen for the identification of a small molecule called kartogenin (KGN) that could induce chondrogenesis by regulating the Core Binding Factor β (CBFβ)- runt-related transcription factor 1 (RUNX1) transcriptional program of multipotent mesenchymal stem cells. This resulted in the repair of degenerative and aging cartilage and could be combined with MSC therapies to gain better outcomes.^162^ Although the rejuvenation of stem cells in OA showed efficacy, the combined effects were still unsatisfactory. In 2024, Li et al. constructed an injectable peptide-hydrogel conjugating a stem cell-homing peptide, PFSSTKT, for carrying plasmid DNA-laden nanoparticles, which could not only recruit BMSCs and increase the chondrogenesis but also release anti-aging protein Klotho via Tanshinon IIA carried by the nanoparticles.^163^ This newly accomplished research suggests the combined usage of MSC modulation and anti-aging interventions, implying the importance and effectiveness of the rejuvenation of stem cells in OA treatment.

#### Limitations and optimization of stem cell therapy of OA

Although current stem cell therapy for OA showed efficacy in animal experiments, limitations of these therapies or proposed strategies to overcome them are still to be assessed for further clinical interventions. For example, fifty clinical studies and 13 systematic reviews/meta-analyses showed that although stem cell therapy presented no adverse reactions in patients and relieved joint pain over time, the knee function was not effectively improved.^164^ As a result, challenges such as low survival rates, immune rejection, and long-term efficacy should be considered together for better clinical outcomes of stem cell therapy for OA.

Firstly, the source of MSCs for OA therapy is of importance for obtaining more effective immunomodulation and differentiation to chondrocytes. Wiggers et al. found that 14 RCTs with a total of 408 patients with KOA received autologous MSC therapy derived from bone marrow, adipose tissue or activated peripheral blood, all of which showed increased clinical outcomes compared with the control. Yet the comparison between different sources of MSCs, especially BMSCs and synovium-derived MSCs (SMSCs), needs to be evaluated.^165^ Autologous BMSCs and ADSCs have been commonly used in RCTs for OA treatment, while SMSCs have gradually been paid attention to due to the ability to differentiate into chondrocytes.^166^ Pan et al. demonstrated that the synovium represents an alternative source of MSCs for patient-derived stem cell therapies comparable to bone-derived MSCs due to the consistent property of a high PDPN/low CD146 profile.^167^ Besides, Shirasawa et al. reported that despite the fact that SMSCs showed similar potential of chondrogenesis with BMSCs from humans, surprisingly, the cartilage pellets derived from SMSCs were larger than those from BMSCs in patient-matched comparisons, indicating SMSCs serve as a more effective source of MSC therapy at the level of chondrogenesis.^168^

What’s more, biomaterials such as hydrogels are proven to improve stem cell viability and delivery efficiency, which could contribute to clinical translational medicine. For example, Li et al. presented a newly developed one-step rapid cross-linking hyper-branched polyPEGDA/HA hydrogel for delivery of MSCs from flushing fluid of joints. Results showed that the MSC/hydrogel composite significantly repaired full-thickness cartilage defects after 8 weeks of implantation in rat model, along with smooth cartilage formation in the area of hyaline cartilage.^169^ Besides, An et al. showed a novel injectable hydrogel system (HAMA@C-MSC) using methylacrylated hyaluronic acid for intraarticular delivery of MSCs, which enhanced chondrocyte regeneration and restored collagen integrity.^170^ Hydrogels could greatly increase the survival of MSCs by effectively preventing cell membrane rupture. Current studies using hydrogels for MSC delivery in OA treatments achieved satisfying results, including fibrin/hyaluronan hydrogel, PEGDA, fibrin MeHA, DNA supramolecular, PEG–hyaluronic acid (HA), Collagen type 1, chondroitin sulfate (CS), collagen and alginate, chondroitin sulfate (CS) and PEG, and chitosan.^171^ Since the improvement of delivery of MSCs to the defected site of cartilage would be a benefit for OA relief, more and more strategies, such as hydrogels and nanoparticles, will emerge for enhancing the survival rate and long-term efficacy of MSCs.

For optimization of direct injection of MSCs, current studies also focus on the usage of MSC exosomes (MSC-Exos) as bioactive factor carriers, which has promising results in cell-free therapy of OA. The sources of MSC-Exos include BMSC, SMSC, ADSC, umbilical cord-derived MSCs (UCMSC), infrapatellar fat pad-derived MSCs (IPFP-MSC), embryonic stem cell- derived MSCs, and urine MSCs. Based on the biological functions, including increasing chondrocyte proliferation and decreasing proinflammatory factors, plenty of studies designed MSC-Exos of different cargos to treat OA, such as miRNA (MiR-92a-3p, MiR-136-5p, MiR-320c, MiR-3960, MiR-125a-5p, MiR-361-5p, MiR-1208, MiR-100-5p, MiR-140-5p, MiR-26a-5p, MiR-155-5p, MiR-129-5p, MiR-338-3p), lncRNA (NEAT1, LYRM4, H19) and circRNA.^172^ Results showed MSC-Exos obviously alleviate OA symptoms via modulation of chondrocytes, immune response, and stem cell senescence,^173^ which is to say, MSC-Exos could serve as a potential alternative to MSCs treatment of OA, which could circumvent immune rejection issues. However, there are still some factors to be considered in clinical intervention, such as the optimal therapeutic dose, source of MSC-Exos, and extraction method of exosomes, frequency of exosome injection.

In summary, the rejuvenation of stem cells can promote chondrogenic potential in the aging microenvironment and is supposed to be a prospective therapy in the future. However, there is still a long way for stem cell therapy to go, as the treatment mechanism, the best cell source, the most appropriate processing method, the most effective dose and delivery procedure, and their efficacy must all be optimized and confirmed.^174^

## Conclusions and perspectives

As the population grows, the research on OA has been paid more and more attention to around the world. New mechanisms in the field of aging have emerged in recent years, with osteoarthritis receiving particular attention due to its unsatisfactory clinical outcomes. Herein, we identified and systematically illustrated the concept of “aging microenvironment” in OA. This concept encompasses immune homeostasis imbalance, cellular senescence and stem cell exhaustion, which are the senescence-related key factors influenced by OA in early stage. Hence, acknowledgment and further investigation of the aging microenvironment could provide precise therapeutic targets in treating early-stage OA.

Different mechanisms of these three factors were discussed about OA, including specific cells involved, their different contributions to OA, and the whole joint microenvironment. Firstly, immune homeostatic imbalance occurs in the early stage of OA. In recent years, inflammation and immune homeostatic imbalance have been thoroughly investigated, and the proinflammatory factors and their function towards senescent cells, stem cell exhaustion, and the extracellular matrix have been partially revealed. In OA, macrophages, T cells, and B cells, as the main functional cells that are mainly resident in the synovium, are activated and differentiate into multiple phenotypes, releasing proinflammatory cytokines and other factors that influence the progression of OA. These types of cells may interact to further amplify the inflammatory response. Also, senescent cells play an essential role in the onset and process of OA caused by aging. The accumulation of senescent cells not only leads to dysfunction of tissue or organs but also produces SASPs, which contain proinflammatory factors as well as matrix metalloprotease. SASPs can degrade the extracellular matrix or influence other components in the aging microenvironment and lead to immune homeostatic imbalance. Besides, the process of aging reveals that stem cells continuously undergo exhaustion. Stem cell exhaustion goes along with cellular senescence and the loss of quantity. The down-regulated self-renewal and function, like migration ability, could decrease the cell turnover in tissue, further impacting the self-renewal ability of tissue and organs. This could further reduce the function of tissue and organs to an integral extent, and it could also bring about a reduction in the extracellular matrix. As for the extracellular matrix, the mechanical microenvironment changes brought about by the degradation of the extracellular matrix were proven to affect the function of the cells inside it.^175,176^ We attempt to shape the concept of OA aging microenvironment by linking the common pathological factors: immune homeostatic imbalance, cellular senescence, and stem cell exhaustion together, and address the prevention and treatment of OA from a comprehensive perspective. However, certain mechanisms of the interactions of these factors are still unclear. Besides, the heterogeneity of aging microenvironment of OA should be paid attention to which informs patient-specific and stage-specific therapeutic strategies. In-depth discussion of the relevant mechanisms will greatly enrich the etiology of OA and contribute to the development of innovative treatment strategies for OA.

At present, there is a gap in the understanding of the aging microenvironment and OA, especially the role of extracellular matrix, mechanical microenvironment and its interaction with immune and aging pathways. Senescent cells secrete various inflammatory mediators and matrix metalloproteinases (MMPs), promoting ECM catabolism and inflammation. Meanwhile, inflammatory factors can also induce cellular senescence and alter ECM composition and properties. The mechanical microenvironment influences cellular senescence and immune responses, with tissue stiffness potentially promoting cellular senescence and modulating immune cell infiltration and function. In terms of the ECM, age-related changes in its proteins, such as glycation, carbamylation, and fragmentation, significantly impact ECM function. Collagen and elastin, the most abundant proteins in the ECM, undergo structural changes due to their long half-life, making them susceptible to various post-translational modifications. These changes compromise the ECM’s ability to provide structural support and mechanical stability, thereby disrupting tissue homeostasis and healthy function. For instance, collagen fragmentation and glycation modifications can lead to matrix stiffness, preventing necessary enzymatic cleavage for remodeling and contributing to OA development. However, the specific mechanisms by which mechanical stimuli regulate ECM metabolism and interact with cellular senescence and immune pathways remain elusive. For example, how mechanical signals are sensed and transduced within the ECM, and how they synergize with biochemical signals to regulate OA development, require further investigation.^177^

Another aspect that warrants investigation is the interfering strategies of OA in terms of regulating aging microenvironments, including therapies and therapeutic targets. Currently, some strategies targeting proinflammatory factors have been implemented for clinical use. Senolytics and senomorphics have been proven to be feasible strategies for controlling the progression of OA, but the specific therapeutic targets still need further research, which calls for deeper investigations of the basic causation of cellular senescence and the precise detection of the pathological and heterogenic genes in OA patients individually. Additionally, stem cell therapies and stem cell rejuvenation therapies are also demonstrated to be effective, but the targets of rejuvenation and the safety and effectiveness of the acquisition of stem cells remain promising areas of future research. Importantly, the possibility and accessibility of clinical translation of the aging microenvironment must be considered to achieve better applications for clinicians. Although senescence-targeting agents (e.g., D/Q combinatorial therapy) and MSC-derived interventions demonstrate efficacy in experimental OA models, their transition to clinical practice encounters substantial translational barriers. Pharmacovigilance frameworks currently lack consensus on standardized monitoring protocols for senescence-clearing agents, particularly regarding potential impacts on quiescent progenitor populations.^178^ Bioethical considerations in regenerative approaches center on somatic cell reprogramming techniques and epigenetic memory erasure during cellular reprogramming.^179^ Besides, economic modeling indicates that senescence-focused treatment protocols may require 1.8–2.3-fold greater initial investment compared to standard symptomatic management, posing challenges for healthcare sustainability.^180^ Consequently, emerging solutions employ multimodal therapeutic architectures, such as concurrent administration of BCL-2 inhibitors with CX3CL1-neutralizing biologics, which are senolytic for eliminating senescent cells and showed 35% greater pain reduction versus monotherapies (NCT04636502) in recent adaptive clinical designs.^181,182^ Stage-adapted therapeutic algorithms now propose TNF-α/VEGF dual blockade for pre-OA metabolic syndrome patients, while late-stage interventions integrate autologous matrix-induced chondrogenesis with targeted senomorphic delivery.^183^ To our acknowledgement, cutting-edge biotechnological innovations are redefining treatment modalities for OA, such as vaccines. For example, a vaccine strategy to generate anti-NGF antibodies was developed for relieving OA pain, since nerve growth factor (NGF) is a driver of pain in OA. Interventional immunization protocols significantly reduced OA pain by incapacitance testing of mice, and analgesic efficacy correlated strongly with maximum antibody concentrations against NGF, demonstrating robust statistical significance.^184^ In recent years, more and more vaccines targeting senescent cells in aging-related diseases have emerged with high efficacy, such as Alzheimer’s disease, type II diabetes, and hypertension. We believe promising emerging technologies could to a significant extent contribute to the early prevention and treatment of OA. What’s more, multidisciplinary approaches, such as Car-T or Car-B therapies in the area of cancer, are also promising methods for precisely targeting senescent cells in OA.

**Fig. 7:**
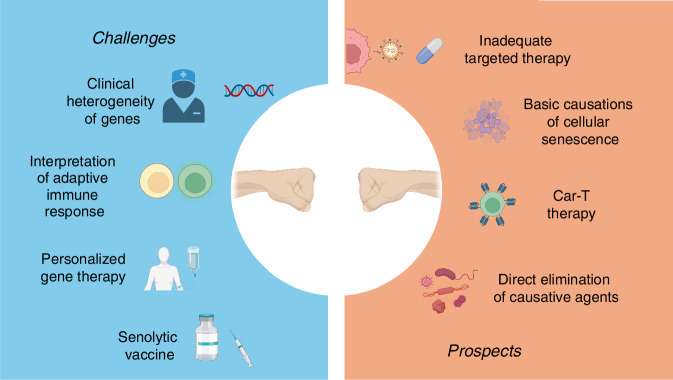
New prospects and challenges in OA. The challenges lie in the interpretation of the adaptive immune response, the basic causality of cellular senescence, etc. Potential new opportunities are the applications of Car-T, senolytic vaccines, etc. Through comprehensive analysis and outlook from different aspects, it is expected that OA can be controlled and treated in the future more effectively

To sum up, we have reviewed the most concerned components affected by aging in OA microenvironment in early stage and discussed related mechanisms and targeted therapies. Although the involvements of immune cells, senescent cells, and stem cells are crucial for OA progression, current investigations still face challenges and barriers to improve targeted and therapeutic efficacy due to the lack of acknowledgment of OA senescence. Hopefully, novel targets combined with conventional approaches for OA treatments provide more possibilities in clinical usage. In conclusion, aging is a natural and integral process for human beings, and osteoarthritis is always associated with the alternation of the aging microenvironment. Hence, by regulating the aging microenvironment, we hope to open the door to entering the joint aging era and to prevent and treat OA in more effective and individualized ways from an integrated view. This is what our review aims to achieve.(Fig. 7)
